# Cell Wall Acetylation in Hybrid Aspen Affects Field Performance, Foliar Phenolic Composition and Resistance to Biological Stress Factors in a Construct-Dependent Fashion

**DOI:** 10.3389/fpls.2020.00651

**Published:** 2020-05-25

**Authors:** Marta Derba-Maceluch, Fariba Amini, Evgeniy N. Donev, Prashant Mohan-Anupama Pawar, Lisa Michaud, Ulf Johansson, Benedicte R. Albrectsen, Ewa J. Mellerowicz

**Affiliations:** ^1^Department of Forest Genetics and Plant Physiology, Umeå Plant Science Centre, Swedish University of Agricultural Sciences, Umeå, Sweden; ^2^Department of Plant Physiology, Umeå Plant Science Centre, Umeå University, Umeå, Sweden; ^3^Biology Department, Faculty of Science, Arak University, Arak, Iran; ^4^Tönnersjöheden Experimental Forest, Swedish University of Agricultural Sciences, Simlångsdalen, Sweden

**Keywords:** *Populus tremula* × *tremuloides*, transgenic trees, field trial, biotic resistance, salicinoid phenolic glucosides, condensed tannins, *HjAXE*, *AnAXE1*

## Abstract

The production of biofuels and “green” chemicals from the lignocellulose of fast-growing hardwood species is hampered by extensive acetylation of xylan. Different strategies have been implemented to reduce xylan acetylation, resulting in transgenic plants that show good growth in the greenhouse, improved saccharification and fermentation, but the field performance of such plants has not yet been reported. The aim of this study was to evaluate the impact of reduced acetylation on field productivity and identify the best strategies for decreasing acetylation. Growth and biological stress data were evaluated for 18 hybrid aspen lines with 10–20% reductions in the cell wall acetyl content from a five year field experiment in Southern Sweden. The reduction in acetyl content was achieved either by suppressing the process of acetylation in the Golgi by reducing expression of *REDUCED WALL ACETYLATION* (*RWA*) genes, or by post-synthetic acetyl removal by fungal acetyl xylan esterases (AXEs) from two different families, CE1 and CE5, targeting them to cell walls. Transgene expression was regulated by either a constitutive promoter (*35S*) or a wood-specific promoter (*WP*). For the majority of transgenic lines, growth was either similar to that in WT and transgenic control (*WP:GUS*) plants, or slightly reduced. The slight reduction was observed in the AXE-expressing lines regulated by the *35S* promoter, not those with the *WP* promoter which limits expression to cells developing secondary walls. Expressing AXEs regulated by the *35S* promoter resulted in increased foliar arthropod chewing, and altered condensed tannins and salicinoid phenolic glucosides (SPGs) profiles. Greater growth inhibition was observed in the case of CE5 than with CE1 AXE, and it was associated with increased foliar necrosis and distinct SPG profiles, suggesting that CE5 AXE could be recognized by the pathogen-associated molecular pattern system. For each of three different constructs, there was a line with dwarfism and growth abnormalities, suggesting random genetic/epigenetic changes. This high frequency of dwarfism (17%) is suggestive of a link between acetyl metabolism and chromatin function. These data represent the first evaluation of acetyl-reduced plants from the field, indicating some possible pitfalls, and identifying the best strategies, when developing highly productive acetyl-reduced feedstocks.

## Introduction

Plant cell walls (lignocellulose) constitute by far the most abundant carbon source on Earth available for the sustainable production of advanced biofuels and “green” chemicals (Bar-On et al., 2018). These products are made through saccharification which converts lignocellulose to fermentable sugars. The industrial saccharification and fermentation processes are challenged by, among other factors, the abundance of acetylation substituents (Jönsson and Martín, 2016) present in most cell wall polymers (Gille and Pauly, 2012; Donev et al., 2018). Dicotyledonous plants, including broadleaf trees (hardwoods), are particularly rich in *O*-acetyl substituents, the majority of which are associated with xylan (Pawar et al., 2013; Pauly and Ramírez, 2018). Biological role of xylan acetylation is not fully understood, but it is known to affect xylan solubility (Gröndahl et al., 2003), susceptibility to enzymatic degradation (Biely et al., 2016), interaction with cellulose (Grantham et al., 2017) and lignin (Giummarella and Lawoko, 2016). On the other hand, there is a considerable variation among different groups of plants in xylan acetylation, and some of them, like conifers, have no acetyl xylan substitution (Pawar et al., 2013). Several attempts have therefore been made to reduce acetyl content in dicotyledon species (Pogorelko et al., 2011; Xiong et al., 2015; Pawar et al., 2016) including hardwoods (Ratke et al., 2015; Pawar et al., 2017a, b; Wang et al., 2020). Based on the performance of greenhouse-grown plants, reductions in *O*-acetylation were found to be well tolerated by plants when the degree of xylan substitution was reduced by 30% or less (Pogorelko et al., 2011; Xiong et al., 2015; Pawar et al., 2016). Moreover, reducing acetylation was found to be one of the most promising strategies for improving plant cell walls for the purposes of saccharification and fermentation (Donev et al., 2018). These results were encouraging, but the performance of such acetylation-reduced lines also needs to be tested in the field.

Field conditions impose both biotic and abiotic stresses on plants, and therefore field performance may be very different from growth observed in the greenhouse (Strauss, 2003). Acetylation-challenged plants in particular could perform differently between these two sets of conditions, since such plants have been shown to react differently to both biotic and abiotic stresses. For example, *Arabidopsis* plants with a mutation in the *TRICHOME BIREFRINGENCY-LIKE 29 (TBL29)* gene encoding a key acetyl transferase involved in secondary wall xylan acetylation (Urbanowicz et al., 2014) were reported to be highly resistant to water deficit and freezing stress, and were thus named *eskimo1 (esk1)* (Xin and Browse, 1998; Xin et al., 2007; Lefebvre et al., 2011; Xu et al., 2014). Plants mutated in *TBL44/PMR5* from the same family are known to be resistant to powdery mildew (Vogel et al., 2004). Similarly, an acetylation deficit caused by mutations in *REDUCED WALL ACETYLATION* (*RWA*) genes that affect acetylation of all cell wall polysaccharides resulted in biotic resistance to biotrophic and necrotrophic fungi in *Arabidopsis* (Manabe et al., 2011; Pawar et al., 2016). Post-synthetic removal of acetic groups from the xylan backbone by transgenic expression of fungal acetyl xylan esterases (AXEs) has been shown to increase resistance to certain pathogenic fungi (Pogorelko et al., 2013; Pawar et al., 2016). Thus deacetylation of xylan appears to lead to better plant resistance to biotic and abiotic stresses. Naturally occurring deacetylation of pectin by the enzyme encoded by *PECTIN ACETYLESTERASE 9* (*AtPAE9*) has been shown to be required for proper basal levels of innate immunity and resistance to aphids (Kloth et al., 2019). A knock out *pae9* mutant with increased rhamnogalacturonan I (RGI) and homogalacturonan acetylation compared to wild-type plants (De Souza et al., 2014) exhibited decreased concentrations of JA, SA, ABA, and IAA, and initial facilitation of cell wall penetration by aphids (Kloth et al., 2019). Although the mechanism by which the cell wall acetylation level is communicated to the plant cell protoplast is at present not known (reviewed by Bacete et al., 2018), it is clear that modifying acetylation can impact plant biotic and abiotic resistance, which are key parameters affecting the field performance of plants.

To assess the field performance of acetylation-reduced plants we tested transgenic hybrid aspen (*Populus tremula* L. × *tremuloides* Michx.) lines in which the acetyl content was reduced by different means. These lines, in which xylan acetylation was post-synthetically reduced, included ones expressing AXEs of fungal origin from two Carbohydrate Esterase families, CE1 and CE5, *Aspergillus niger* AXE1 (*An*AXE1) and *Hypocrea jecorina* AXE (*Hj*AXE), respectively (Ratke et al., 2015; Pawar et al., 2017b; Wang et al., 2020). *AnAXE1* and *HjAXE* expressing lines grown in a greenhouse environment developed as well as wild type, and had superior saccharification properties. The lines in which acetyl content was reduced due to deficiencies in the biosynthetic acetylation machinery were those with reduced expression of native *REDUCED WALL ACETYLATION* (*RWA*) genes (Pawar et al., 2017a). The latter lines exhibited similar reductions in acetylation and improved saccharification properties, with good growth in the greenhouse, as the lines in which the xylan was post-synthetically deacetylated. Using these lines we were able to compare the impacts of reducing acetylation during the biosynthesis of xylan in the Golgi by suppressing *RWA* genes with those where the reduction was achieved post-synthetically in cell walls by expressing the fungal enzymes *An*AXE1 and *Hj*AXE targeted to the apoplast. We also addressed the question of the promoter to be used for genetic engineering. We compared the effects of the same transgenes expressed from either constitutive *35S* or wood-specific *WP* (Ratke et al., 2015) promoters. Finally, we compared the effects of two fungal enzymes belonging to the different CE families. We monitored growth over five years, and determined foliar biotic damage and foliar concentrations of phenylpropanoid compounds, which are indicators of stress induction and stress resistance (Dixon and Paiva, 1995; Papazian et al., 2019). In *Populus* spp., biotic stress has commonly been associated with levels of condensed tannins (CTs) (Bandau et al., 2015; Lindroth and Madritch, 2015; Lindroth et al., 2015) and salicinoid phenolic glucosides (SPGs) (Albrectsen et al., 2010; Robinson et al., 2012; Lindroth and St. Clair, 2013; Lindroth and Madritch, 2015), and these phenylpropanoid compounds are often related to environmental stress responses and performance (Lindroth et al., 2011; Robinson et al., 2012; Keefover-Ring et al., 2014; Bandau et al., 2015; Decker et al., 2016). We therefore measured foliar concentrations of these compounds in acetylation-compromised aspen lines. This is the first analysis of the field performance of plants with reduced acetylation.

## Materials and Methods

### Biological Material

Hybrid aspen (*Populus tremula* L. × *tremuloides* Michx.) clone T89 was used as wild-type and all transgenic lines were made in this genetic background. Transgenic lines initially tested in the greenhouse included those expressing *35S:An*AXE1 (Pawar et al., 2017b), *35S:HjAXE* and *WP:Hj*AXE (Ratke et al., 2015; Wang et al., 2020), as well as lines with RNAi constructs targeting hybrid aspen *RWA-C* and *RWA-D* genes, denoted *35S:RWA-CD* (previously called *35S::CD-RWA RNAi*), and all four *RWA* genes, denoted *WP:RWA-ABCD* (previously called *pGT43B::RWA-ABCD RNAi*) (Pawar et al., 2017a). Two lines expressing a β-glucuronidase (GUS) gene under the control of the *WP* promoter (Ratke et al., 2015) were used as transgenic controls. Additional lines were generated that expressed *WP:AnAXE1* using the *pK-pGT34B-GW* destination vector (Ratke et al., 2015) and the *AnAXE1* cDNA as previously described (Pawar et al., 2017b). Each construct was represented by two to four lines selected from among approx. 20 independent lines, as described previously, based on the strength of transgene expression and superiority of greenhouse performance with regard to growth and saccharification properties (as described by Ratke et al., 2015; Pawar et al., 2017a, b; Wang et al., 2020), with the exception of *WP:AnAXE1*. The *WP:AnAXE1* lines were selected based on the strength of transgene expression in plants cultivated *in vitro*, as determined by RT-PCR analysis (Supplementary Figure S1). A list of the lines and constructs is given in Table 1.

**TABLE 1:** Lines included in the field trial analysis.

**Construct**	**Lines**	**References**
*35S:RWA-CD*	10, 21, 22	Pawar et al., 2017a
*WP:RWA-ABCD*	11, 15	Pawar et al., 2017a
*35S:AnAXE1*	4, 8, 17	Pawar et al., 2017b
*WP:AnAXE1*	1, 5, 8, 10	This paper, Supplementary Figure S1
*35S:HjAXE*	9, 13, 22	Ratke et al., 2015; Wang et al., 2020
*WP:HjAXE*	11, 14B, 14C	Ratke et al., 2015; Wang et al., 2020
*WP:GUS*	25, 27	Ratke et al., 2015

### Field Trial Establishment and Experimental Design

Transgenic trees were propagated *in vitro* at the Umeå Plant Science Centre transformation facility in Umeå, Sweden (Nilsson et al., 1992) and transplanted into soil for one month of acclimatization in the greenhouse in early spring 2014. Then, with permission from the Swedish Board of Agriculture (DNR. 4.6.18-761/14), the trees were moved to sheltered outdoor premises in Umeå for a two-week hardening period, before translocation to the field site (ca 1000 km south of Umeå) in Våxtorp, Laholm community, Sweden (56.42°N, 13.07°E). Between August 4 and 8, 2014, the trees were planted in the field, with a 3 m spacing, on abandoned farm land fenced according to the requirements for genetically modified plants. In total, 636 trees included in the current analysis, along with other trees not analyzed here, were arranged in a 14 block design, with two trees of each transgenic line randomly distributed within each block along with four wild-type (WT) trees (Supplementary Figure S2). For weed control, the field was harrowed twice a year during the first two years following planting and grass was mowed twice a year during subsequent years. All biosecurity and safety procedures for field trials with transgenic plants required by the Swedish Board of Agriculture have been adhered to.

### Histochemical Analysis of GUS Expression

The stability of GUS gene expression was investigated in July of year four (2017) during the period of active cambial growth. The basal part of a two-year-old branch was hand sectioned and the sections were prefixed in acetone for 30 min, washed with water, placed in the reaction solution (1 mM X-GlcA (5-bromo-4-chloro-3-indolyl β-D-glucuronide), 50 mM Na-phosphate buffer pH 7, 0.1% (v/v) Triton, 1 mM K_3_[Fe(CN)_6_], 1 mM K_4_[Fe(CN)_6_]), and incubated for 3 days in the dark at room temperature. Sections were then fixed in FAA (50% ethanol, 5% formaldehyde, 10% acetic acid, all v/v) overnight followed by clearing and dehydration in an ethanol series. Samples were rehydrated, mounted in 50% (v/v) glycerol, and imaged with a Zeiss Axioplan 2 microscope using a 40× objective. Micrographs were taken with an Axiocam HRc camera and Axiovision V 4.8.2 Software (Carl Zeiss Light Microscopy, Göttingen, Germany). Images were combined into panoramas covering the section from bark to pith using the program Adobe Photoshop CS6.

### Periodic Growth and General Damage Assessment

Growth parameters (plant height and root collar diameter) were assessed at the end of each growth season. Final tree height and diameter were also measured in June 2018, before harvesting. The height was assessed with a measuring stick and the stem diameter with a caliper (3 cm above ground level). Stem volume was calculated as: *V* = 1/3π*R*^2^*H*, where *R* – stem radius, *H* – stem height.

Standard assessments (Nilsson and Örlander, 1999) of damage were conducted four times a year; they included cause of damage (fungi, frost, drought, waterlogging, rodents, herbivores, insects, vegetation, unknown) and severity on a six-level scale (0 = undamaged, 1 = slight damage, 2 = uncertain or moderate damage, 3 = severe damage, 4 = life-threatening damage, and 5 = dead).

### Detailed Biotic Stress Assessment and Leaf Collection

A detailed assessment of leaf damage was performed in July 2017 during the fourth growing season, according to previous methodology (Albrectsen et al., 2010; Robinson et al., 2012). Chewing damage by arthropods (chewing) was assessed as percentage of chewed leaves in the canopy. Evidence of other types of damage was scored in terms of presence (1) or absence (0). This was done for damage caused by arthropods including aphids, miners, gall-producing organisms, and pathogens including rust (*Melampsora spp.*) and venturia (*Venturia spp.*). Symptoms of chlorosis, necrosis, and hypersensitive response (HR) were recorded in the same way.

### Assessment of Traits Related to Architecture and Leaf Chemical Profiling

Architectural traits, chlorophyll index, and leaf CT contents were assessed for a subsample consisting of 50% of trees having superior height selected from each transgenic line and wild type. The rationale for stratifying the samples by height rather than randomly selecting 50% of trees from each line was to avoid those trees that had been damaged by planting, field work or other types of random disturbance.

#### Architectural Traits

Branching was assessed according to Luquez et al. (2008). The apical dominance was scored on a scale from 0 to 8 as follows: clear leader (8)/good recovery after apex damage in 2017 (7)/good recovery after apex damage in 2016 (6)/good recovery after apex damage in 2015 (5)/main shoot lost in 2015 (4)/main shoot lost in 2016 (3)/main shoot lost in 2017 (2)/bushy growth (1)/dwarf (0).

#### Chlorophyll Content

Chlorophyll content was measured in fully developed leaves with no visible damage, collected from the upper part of the main stem between June 26 and 30, 2017, using a CCM-200 plus (Opti-Science, Huston, United States). A mean value from 18 measurements per tree was obtained (six leaves per tree and three measurements per leaf). The same leaves were collected for metabolite analyses and dry weight assessment. They were immediately frozen on dry ice and freeze-dried before being transported to Umeå for weighing, grinding and metabolic profiling.

#### Condensed Tannins

Six freeze-dried leaves per tree were ground together to a powder. Foliar CT contents were assessed based on the acid-butanol method of Porter et al. (1986). In short, 10.0 ± 2.0 mg leaf powder (exact weight) was extracted with 800 μl of a mix of acetone and 10 mM ascorbic acid solutions in a 70:30 (v:v) ratio, mixed by vortexing, sonicated, and centrifuged for 5 min at 3500 rpm on a bench top centrifuge. The absorbance of the extract (150 μl of supernatant) at 550 nm was measured with a spectrophotometer (Hitachi U-5100 UV/VIS, Hitachi High-Technologies, Tokyo, Japan). Results were compared to a standard curve of procyanidin B2 (C_30_H_26_O_12_, Sigma-Aldrich^®^, St. Louis, MO, United States) and recalculated to give mg/g (d.w.) leaf powder.

#### Metabolite Analysis

Four trees per line were randomly selected from the set used for leaf CT determination. Ultra high performance liquid chromatography (UHPLC) with UV and electro-spray ionization time-of-flight mass spectrometry (ESI-TOF/MS) detectors was used as described by Abreu et al. (2011) and Keefover-Ring et al. (2014). In short, 10.00 ± 1.00 mg of ground leaf material was extracted in 1 ml of cold (4°C) methanol: chloroform: water, 60:20:20 (v:v:v), with deuterated SA as an internal standard. After centrifugation, 200 μl of the extract supernatant was dried in a speedvac. Before analysis, the samples were reconstituted with 20 μl of methanol and 20 μl of a 0.1% v/v aqueous formic acid solution. Compounds in the reconstituted plant extracts were separated on a C18 UPLC^TM^ column (2.1 × 100 mm, 1.7 μm) and analyzed by an Acquity photodiode array detector coupled in line with a LCT Premier TOF/MS (all from Waters, Milford, MA, United States) as described by Abreu et al. (2011).

The MassLynx 4.1 software package (Waters Corp.) was used to extract single ion chromatograms (±0.15 exact mass unit) using the QuanLynx module to search for known and theoretical phenylpropanoids (using deprotonated ([M−H]^–^) and formate adduct ([M−H+FA]^–^) ions). QuanLynx software was used to obtain peak areas that were normalized with respect to internal standard peak area and sample weight; as described in Abreu et al. (2011) and Keefover-Ring et al. (2014). The phenylpropanoids salicortin, tremulacin, salicin, tremuloidin, salicyloylsalicin, HCH-salicortin, 2′-(E)-, and 2′-(Z)-cinnamoylsalicortin were determined using retention times and molecular weight information for purified standards. Other compounds (2′-acetylsalicin, 2′-acetylsalicortin, acetyl tremulacin, HCH-2′-acetylsalicortin, HCH-tremulacin, and arachidonic acid) were tentatively identified based on LC-MS molecular weights and defragmentation patterns.

### Statistical Analyses

All analyses were performed using the software package JMP 14.0.0 2018 (SAS Institute Inc.). The consequences of decreased acetylation for field performance were analyzed for a total of 19 growth related traits, 14 biotic stress related traits, and 23 foliar defense related chemicals (mainly phenylpropanoids). Effects of individual lines were tested by a one-way ANOVA with “line” used as fixed effect (Supplementary Tables S1, S2). Similarity of individual lines to WT was evaluated by a Dunnett’s test, and consistent line effect within a construct were assessed by a contrast analysis (all lines for a given construct versus WT). These results were used to identify cases of reproducible construct effects among different transgenic lines.

A nested-ANOVA model design was used to answer questions about the impact of construct on phenotypic trait expression. “Line” nested in “construct” and block (random, considered when possible) effects were included (Supplementary Tables S3, S4). A multiple comparison Tukey test was used for cross-comparisons among different constructs.

Impacts of the promoter and the transgene (fixed effects) were analyzed by a two-way ANOVA with an interaction (Supplementary Table S6), and comparison of deacetylation strategy was carried out by a nested ANOVA model with “construct” nested in “pre- or post-synthetic strategy” and “line” nested within “construct” (Supplementary Table S7), all used as fixed effects.

## Results

### Field Growth Analysis Identified Three Lines With Anomalies

**FIGURE 1:**
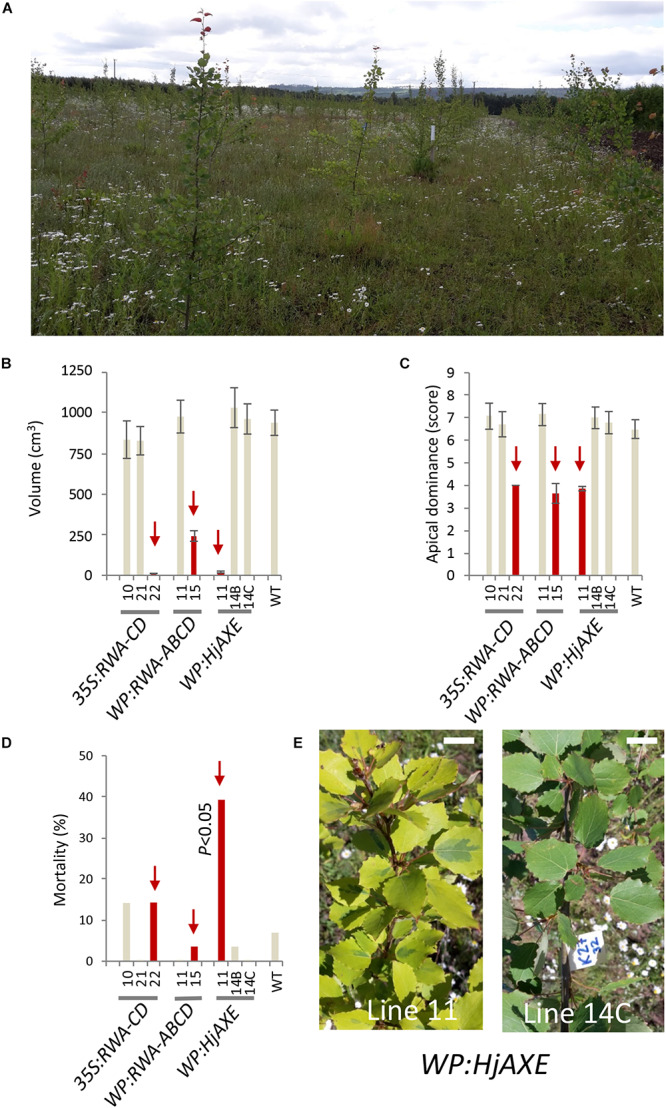
Field testing revealed striking phenotypes in three out of 20 lines tested, effects which could not be related to transgenes. Overview of the field trial in July 2017 (fourth year) **(A)** and the corresponding data for anomalous lines: stem volume **(B)**, apical dominance **(C)** and mortality within the lines **(D)**. Lines marked in red showed aberrant morphology compared to other lines with the same construct and to WT. Line 11 with the *WP:HjAXE* construct had higher mortality than all other lines, and exhibited a variegated phenotype **(E)** not seen in other lines carrying this construct. Scale bar in E – 2 cm. Data in panels **(B)** and **(C)** are means ± SE.

Uniform growth and a stable survival rate of close to 100% (86–100%) characterized the majority of the transgenic lines throughout the period of testing at the field site (Figure 1A and Supplementary Table S1). However, three out of 20 transgenic lines for three different constructs (namely line 22 for construct *35S:RWA-CD*, line 15 for construct *WP:RWA-ABCD*, and line 11 for construct *WP:HjAXE*) exhibited distinct deviations from the general growth pattern. These lines were dwarf, reaching only 1, 26, and 2% of WT stem volume, respectively, and their apical dominance was reduced compared to that of WT and other lines with the same construct (Figures 1B,C and Supplementary Table S1). Line 11 of *WP:HjAXE* had significantly higher mortality than the other transgenic lines and WT (Figure 1D), and approx. 50% of trees of this line showed a striking variegated phenotype that suggested genomic instability (Figures 1D,E). None of these phenotypes were seen in greenhouse trials (Ratke et al., 2015; Pawar et al., 2017a, b; Wang et al., 2020). The three dwarf lines were also more affected by hare browsing and multiple injuries than the other lines or WT trees (Supplementary Table S1).

Since other lines with the *WP:HjAXE* and *35S:RWA-CD* constructs did not exhibit dwarf phenotypes, and moreover since line 11 of *WT:HjAXE* had lower transgene expression levels than the other lines carrying this construct (Wang et al., 2020), we do not consider these anomalies to have been caused by the respective transgenes. Rather these phenotypes should be attributed to mutations induced either by random transgene insertions in the genome or by somaclonal variation. However, the *WP:RWA-ABCD* construct was represented by only two lines that had very different phenotypes (Figures 1B–D and Supplementary Table S1). It was therefore not possible to infer any true construct effect from the data and both these lines were subsequently omitted from analyses testing construct effects. Thus, the subsequent analyses evaluate the effects of five constructs: *35S:RWA-CD*, *35S:AXE1*, *35S:HjAXE*, *WP:AnAXE1*, and *WP:HjAXE*, with the anomalous lines removed from analyses.

### Effects of Constructs on Tree Growth and Development

**FIGURE 2:**
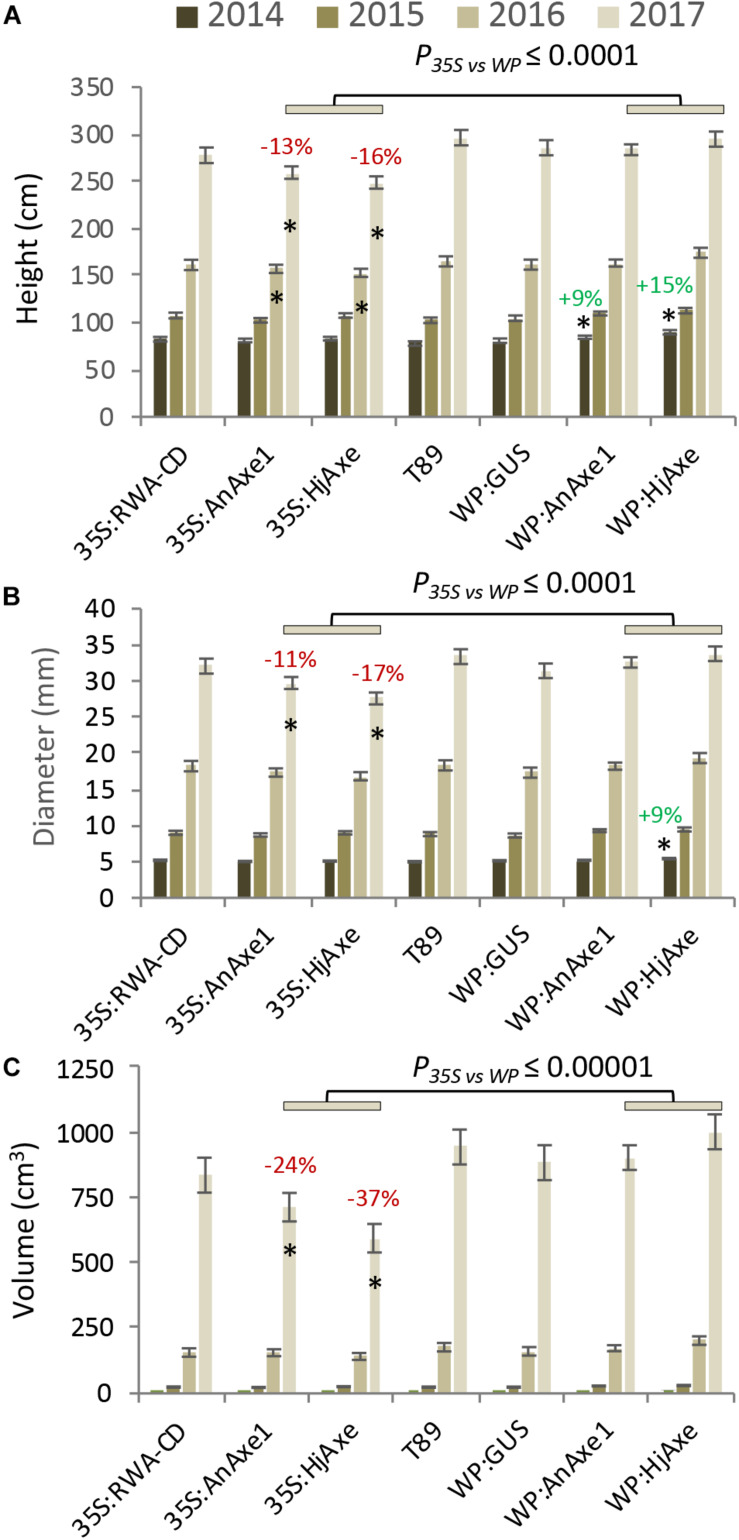
Effects of different types of genetic modification on growth of transgenic lines during a four-year field trial. Height **(A)**, diameter **(B)**, and stem volume **(C)**. Means and SE, stars indicate means significantly different from WT, post-ANOVA contrast, *p* ≤ 0.05). *P*-value indicates significance of the promoter effect based on a two-way ANOVA (Supplementary Table S6).

Stem growth parameters (height and diameter) for the different constructs are shown in Figure 2 and Supplementary Tables S2, S3. Trees expressing *WP:AXE1* and *WP:HjAXE* were slightly bigger than WT in the first year, but this advantage disappeared during the subsequent years. In contrast, those with two other constructs, *35S:AnAXE1* and *35S:HjAXE*, showed a small reduction in height and /or stem diameter after three and four years of growth (Figures 2A,B), resulting in a decrease in stem volume in the fourth year of 24 and 37%, respectively, as compared to WT (Figure 2C).

**FIGURE 3:**
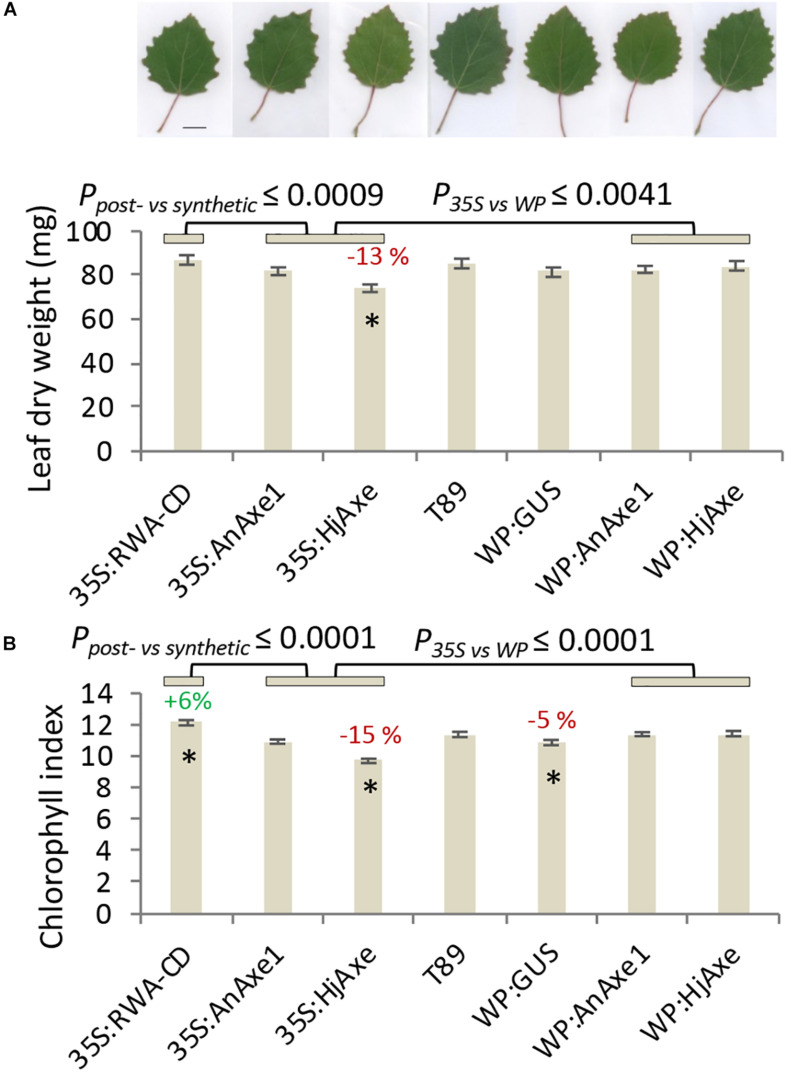
Effects of different types of genetic modification on leaf traits. Leaf dry weight with representative images of leaves, size bar = 1 cm **(A)**. Leaf chlorophyll index **(B)**. Means and SE, stars indicate means significantly different from WT, post-ANOVA contrast, *p* ≤ 0.05). *P*-value indicates significance of the promoter effect based on a two-way ANOVA (Supplementary Table S6) and synthetic vs. post-synthetic xylan modification (Supplementary Table S7).

Shoot apical dominance and the branching pattern did not show any construct-related effects (Supplementary Tables S2, S3). Leaf dry weight and chlorophyll content were reduced in *35S:HjAXE* expressing trees as compared to WT, but their leaf morphology did not change (Figure 3). Interestingly, the chlorophyll content was slightly increased in *35S:RWA-CD* (Figure 3); this was not observed in any of the *WP:RWA-ABCD* lines (Supplementary Table S1). Unexpectedly, a small decrease in chlorophyll content was observed in *WP:GUS* lines.

To assess the suitability of the *35S* and *WP* promoters and the two *AXE* transgenes, *AnAXE1 and HjAXE* for transgenic expression, we analyzed the growth traits of a subset of transgenic lines (*35S:AXE1*, *35S:HjAXE*, *WP:AXE1*, *WP:HjAXE*) by a two-way ANOVA with promoter and transgene as fixed effects (Supplementary Table S6). This comparison revealed a positive effect of *WP*, compared to the *35S* promoter, on stem height, diameter, volume, and chlorophyll index (Figures 2A–C, 3B and Supplementary Table S6). In contrast, there was hardly any effect of the transgene (*AnAXE1* vs. *HjAXE*) on growth, whereas the chlorophyll index was decreased in *HjAXE* compared with *AnAXE1* but only when combined with the *35S* promoter (Figure 3B and Supplementary Table S6).

**FIGURE 4:**
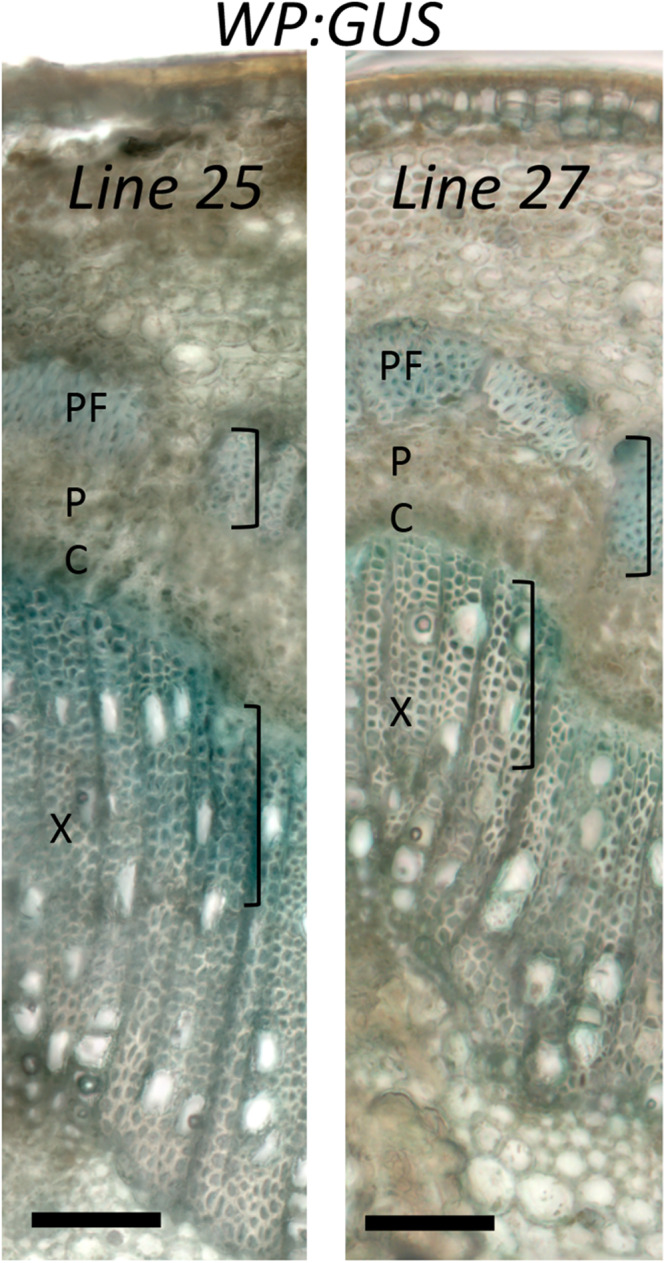
Activity of wood-specific promoter (*WP*) in trees grown in the field. Histochemical β-glucuronidase analysis of a branch with one-year old cambium of *WP:GUS* trees (lines 25 and 27) in the fourth growing season in July, during the active wood production period. X – secondary xylem; P – secondary phloem, PF – phloem fibers; C-vascular cambium. Activity is seen in cells depositing secondary cell walls (marked with brackets). Scale bar = 100 μm.

Specificity of *WP* activity has been previously tested in greenhouse conditions (Ratke et al., 2015). We therefore investigated whether its activity and expression pattern are maintained in field conditions, using histochemical β-glucuronidase analysis of two *WP:GUS* lines during the fourth growing season. The test was carried out on branches with 1- and 2-year old cambia. In both samples and both lines, β-glucuronidase expression was detected in differentiating secondary xylem cells and secondary phloem fibers and sclereids depositing secondary walls (Figure 4 and Supplementary Figure S3). This expression pattern was consistent with that previously observed in the same lines in the greenhouse (Ratke et al., 2015). We conclude that the activity and specificity of *WP* was not altered in the field.

We also investigated whether growth was differentially affected in plants in which xylan acetylation was modified during biosynthesis in the Golgi compared to plants in which post-synthetic xylan deacetylation was implemented (denoted *post-* vs. *synthetic* comparison). To answer this question, the lines with *RWA-CD* RNAi suppression driven by the *35S* promoter were compared with those where fungal *AXE*s were driven by *35S* using ANOVA (Supplementary Table S7). For these constructs, growth, assessed by stem height, diameter and volume, and leaf dry weight, was scarcely affected by the engineering strategy used; only the final height and diameter (measured in the middle of the fifth growing season) were slightly reduced in lines with the post-synthetic deacetylation strategy compared to those with synthetic reduction (Supplementary Table S7). This growth inhibition was preceded by a decrease in leaf dry weight and chlorophyll contents in the fourth year in lines with post-synthetic reduction (*35S:AnAXE1* and *35S:HjAXE*) (Figure 3).

### Stress Response Traits

**FIGURE 5:**
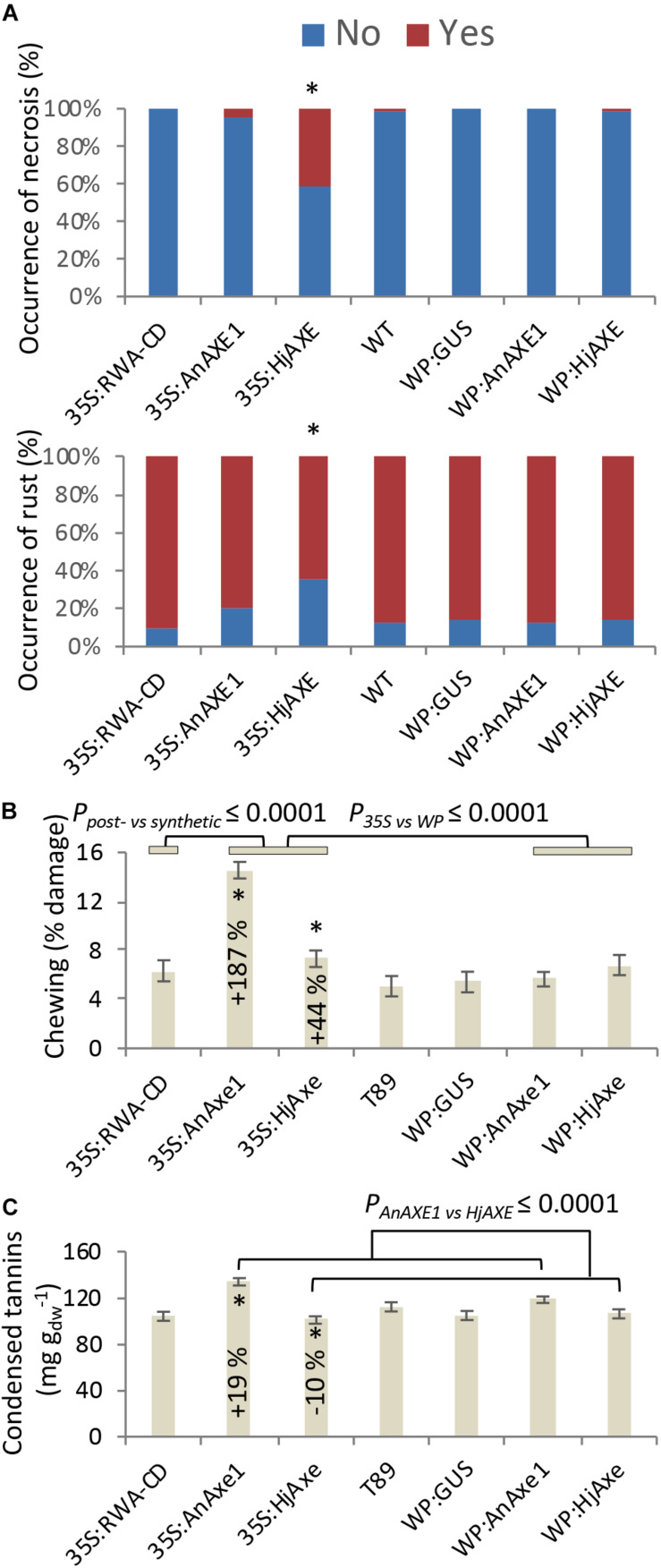
Effects of different types of genetic modification on biotic stress responses. Instances of leaf damage recorded in the 2017 survey that showed significant effects of “construct.” **(A)** Necrosis and rust. Constructs producing significantly different distributions are marked with *. Details of statistical analysis are provided in Supplementary Table S5. **(B)** Chewing damage. **(C)** Condensed tannin contents. Data in B and C are means and SE, stars indicate means significantly different from WT (post-ANOVA contrast, *p* ≤ 0.05). *P*-value indicates significance of the transgene or promoter effect based on a two-way ANOVA (Supplementary Table S6) and synthetic vs. post-synthetic xylan modification strategy (Supplementary Table S7).

Stress-related responses were recorded regularly throughout the four-year field test period, and detailed mapping of necrosis, rust and chewing symptoms was additionally conducted in 2017 simultaneously with collection of leaf material for phenolic profiling. Some of these traits were related to construct identity and, in particular, to the choice of promoter (Figure 5 and Supplementary Tables S2, S3, S5–S7). Necrosis was generally elevated for *35S:HjAXE* plants, potentially at the expense of rust symptoms (Figure 5A), which were generally reduced on plants belonging to the same construct. Moreover, both kinds of fungal AXE transgenes under the *35S* promoter (Figure 5B) suffered from an increase in the extent of chewing symptoms, with damage being increased by 187% (*35S:AnAXE1*) and 44% (*35S:HjAXE*) compared to wild type plants. Synthetic acetylation reduction (*35S:RWA-CD*), on the other hand, had no impact on chewing damage (Figure 5B).

Levels of phenolic compounds that are often associated with damage risk varied in a genotype-related way. For example, salicortin and HCH-acetyl-salicortin were elevated in *35S:RWA-CD* trees (Table 2). *35S:AnAXE1* and *35S:HjAXE* lines also expressed higher levels of certain SPGs, such as OH-tremuloidin, whereas acetyl-tremulacin and *p*-coumaric acid were greatly elevated in *35SAnAXE1*. Salicylic acid, on the other hand, was reduced in *35S:HjAXE* trees. Thus, use of the constitutive 35S promoter led to more alterations in concentrations of SPGs compared with transgenics created with the help of the *WP* promoter, which did not deviate significantly from the wildtype (WT, Table 2).

**TABLE 2:** Metabolite (mostly phenylpropanoid) profiles affected by genetic transformation (construct) based on nested ANOVA.

	***35S:RWA-CD***	***35S:AnAXE1***	***35S:HjAXE***	**WT**	***WP:GUS***	***WP:AnAXE1***	***WP:HjAXE***
Acetyl-salicortin	458047 *a* ± 27307	403962*ab* ± 22296	332471*b* ± 21540	387394*ab* ± 38618	419924*ab* ± 27307	408180*ab* ± 20098	442228*a* ± 27307
Acetyl-tremulacin	2182*b* ± 954	109067a*±779	2293*b* ± 753	3158*b* ± 1349	2861*b* ± 954	3206*b* ± 702	2950*b* ± 954
Benzoic acid	9397*ab* ± 683	9340*a* ± 558	6842*b* ± 539	8803*ab* ± 966	7745*ab* ± 683	8337*ab* ± 503	7808*ab* ± 683
C*a*techin	18107820*b* ± 507156	20517646a*±414091	19323366*ab* ± 400050	18648679*ab* ± 717226	18937764*ab* ± 507156	19594648*ab* ± 373256	18383082*b* ± 507156
Cinnamoyl-salicin	12097*ab* ± 987	11944*ab* ± 806	10358*b* ± 779	11808*ab* ± 1396	13966*a* ± 987	12987*ab* ± 727	14650*a* ± 987
HCH-Ac-salicortin	30552a*±2279	15154*b* ± 1861	22385*ab* ± 1798	22058*ab* ± 3223	25912*a* ± 2279	23954*a* ± 1677	25120*a* ± 2279
HCH-salicortin	2027209a*±117742	1941761*a* ± 96136	1590870*a* ± 92876	1819946*a* ± 166513	1814078*a* ± 117742	1891767*a* ± 86656	1690340*a* ± 117742
HCH-tremulacin	779420*ab* ± 54671	901451*a* ± 44639	702145*b* ± 43125	856067*ab* ± 77317	854546*ab* ± 54671	850986*ab* ± 40237	773174*ab* ± 54671
OH-tremuloidin	308376*bc* ± 22013	379038ab*±17973	404189a*±17364	295890*abc* ± 31131	295881*bc* ± 22013	312900*bc* ± 16201	285702*c* ± 22013
*p*-coumaric acid	72598*b* ± 5669	117312a*±4628	69830*b* ± 4471	80959*b* ± 8017	83148*b* ± 5669	76046*b* ± 4172	81188*b* ± 5669
Salicoyl-tremuloidin	3393*ab* ± 536	3518*a* ± 438	1419*b* ± 423	2618*ab* ± 758	3566*a* ± 536	3503*a* ± 394	4019*a* ± 536
Salicortin	31708303*±1359824	27844354 ± 1110292	27399864 ± 1072644	26846382 ± 1923082	27934434 ± 1359824	30052206 ± 1000803	30601384 ± 1359824
Salicylic acid	1202017*a* ± 23487	1155301*ab* ± 19177	1085672b*±18527	1179529*ab* ± 33216	1214922*a* ± 23487	1174446*a* ± 17286	1160759*ab* ± 23487
Arachidonic acid	5687b*±252	6418*ab* ± 205	5929b*±198	7235*a* ± 356	6013ab*±252	6476*ab* ± 185	6108ab*±252
							

Means ± SE and Tukey’s test data (letters) are extracted from Supplementary Table S4. Stars * and bold indicate means significantly different from WT (post-ANOVA contrast *P* < 0.05, Supplementary Table S2).

Catechin (the precursor of CTs) was elevated in *35S:AnAXE1*, which also had higher levels of CTs (Figure 5C). The elevated CT level characteristic of genotype *35S:AnAXE1* was accompanied by very much higher levels of damage caused by chewing herbivores, whereas a general decrease in CT levels for *35S:HjAXE* was also associated with elevated herbivory (by 44%). That the two fungal *35S* transformations were associated with varying responses in terms of CTs, but at the same time had similar susceptibility to herbivores, indicated a lack of any general relationship between CT levels and the risk of chewing damage.

In addition to SPGs, we monitored the foliar levels of arachidonic acid, a polyunsaturated fatty acid (20:4 Δ5,8,11,14) regulating different physiological and stress responses that is found in some plant species including poplars (Groenewald and van der Westhuizen, 1997). It has been shown to trigger different plant stress responses and induce resistance to fungal pathogens (Savchenko et al., 2010). Interestingly, the levels of arachidonic acid were decreased compared to WT in lines with all constructs except for those containing *AnAXE1* (Table 2). This suggests that the quality of lines carrying *AnAXE1* constructs is potentially superior compared to that of other transgenic lines with regard to biotic stress resistance.

## Discussion

### Good Growth and Field Performance of Lines Carrying Constructs Targeting Acetylation

We have tested for the first time the field performance of trees with transgenically reduced cell wall acetyl content. We found that, in general, apart from those lines with abnormal phenotypes, the reduction of acetylation in cell walls did not lead to either major detrimental effects or significant growth stimulation in the field (Figures 2, 3). The level of reduction in acetyl content for the lines previously tested varied between 10 and 16% in *WP:HjAXE* (lines 11, 14B, 14C; Wang et al., 2020), and 13 and 16% in *35S:AnAXE1* (lines 4, 8, 17; Pawar et al., 2017a), and it was 20% in *35S:RWA-CD* (line 10; Pawar et al., 2017b). The reductions in acetyl content in *WP:AnAXE1* lines have not been analyzed, but based on previous comparisons between *35S* and *WP*-driven transgenes (Ratke et al., 2015), and the documented observation that the specific activity of the WP is stable (Figure 4), we expect a slightly stronger effect with *WP:AnAXE1* than with *35S:AnAXE1*. Thus, the mild reductions in cell wall acetylation levels (by 30% or less) are well supported by plants both, in the field (Figure 2), and in the greenhouse conditions (discussed in Pawar et al., 2017b; Donev et al., 2018).

### Better Field Performance of WP Compared to 35S Promoter

Analyses of growth and biotic stress responses in lines harboring *35S*- and *WP*-driven fungal AXEs point to a clear advantage offered by the specific transgene expression achieved with the *WP*. Both height and diameter growth were reduced in *35S* lines compared to *WP* lines, and the stem volume was reduced by as much as 21% and 41% in, respectively, *35S:AnAXE1* and *35S:HjAXE* compared to the corresponding *WP* constructs (Figure 2). These growth penalties in *35S* lines were seen only in the field; these lines did not exhibit growth defects in the greenhouse (Ratke et al., 2015; Pawar et al., 2017b; Wang et al., 2020). One factor which could have contributed to the growth penalty in the field is the alteration in interaction with herbivores as revealed by the more extensive foliar chewing damage (Figure 5B). The increase in susceptibility to herbivores is likely to be due to metabolic changes in the leaves in *35S* plants caused by AXE activity, changes which are largely avoided when *WP* targets the transgene expression to the developing wood. The fungal AXEs targeted to cell walls are expected to hydrolyze acetyl esters liberating acetic acid, thus changing the pH of the leaf, and since acetic acid can cross membranes in uncharged form, the reaction could contribute to the biosynthesis of acetyl-CoA. This compound is used in a variety of reactions, including the TCA cycle, glyoxylate cycle, lipid biosynthesis, mevalonate pathway, and it is considered an energy-status marker for a eukaryotic cell (Cai and Tu, 2011). It is therefore perhaps not surprising that ectopic manipulation of acetyl-CoA pools can have far-reaching consequences, and our results demonstrate that restricting the transgenic modification to specific tissues, such as developing wood, can prevent or attenuate such undesirable side-effects.

### Synthetic Versus Post-synthetic Deacetylation Strategies

Assuming that the *RWA* genes encode Golgi-localized acetyl-CoA transporters (Gille and Pauly, 2012; Pauly and Ramírez, 2018), cytosolic accumulation of acetyl-CoA is expected when expression of these genes is suppressed. Apoplastic AXE expression which results in high acetic acid levels in the apoplast might eventually result in a similar outcome, assuming that acetic acid diffuses via membranes and is converted to acetyl-CoA by acetyl-CoA synthases. Even though the cytosolic accumulation of acetyl-CoA might be similar with the synthetic and the post-synthetic strategy, these two strategies might lead to differences in cell wall polymer structures. For example, increased glucuronosylation of xylan is expected when the xylan acetylation machinery is suppressed since the two processes apparently compete for the same substrate (Chong et al., 2014; Xiong et al., 2015; Grantham et al., 2017). Post-synthetic deacetylation might be also more specific toward a targeted polymer – xylan – in particular, *Hj*AXE, which was used in this study for the post-synthetic modification, has documented xylan specificity (Koutaniemi et al., 2013).

Comparisons of the phenotypic effects of synthetic versus post-synthetic strategies (both using the *35S* promoter) revealed that some leaf-related traits were affected. Leaf weight, chlorophyll content, and chewing resistance appeared to be lower in the case of post-synthetic modification (Figures 3, 5). Many SPGs were also affected by the deacetylation strategy (Supplementary Table S7). These foliar changes were not matched by stem growth during the first four years in the field, but in the final (fifth) year, both stem diameter and stem height were somewhat reduced by the post-synthetic modification strategy compared to the synthetic one (Supplementary Table S7). The lines available only allowed us to draw conclusions about the pre- vs. synthetic strategy in the case of ectopic modification using the *35S* promoter. It would be interesting to investigate whether the same conclusion applies to modification targeted specifically to developing wood.

### Variability in Foliar Phenolics and Resistance Properties of the Transgenic Lines

Although the trees in this study were never exposed to an outbreak of severe attack by a particular herbivore or pathogen, the relatively low and variable relationships in the field between various kinds of biotic stressors and genotypes confirmed that the transgenic procedure in itself is unlikely to be associated with any systematic impact on surrounding organisms and *vice versa* (Strauss, 2003).

Leaf CTs of natural aspen populations are strongly tied to genotype (Lindroth et al., 2011; Robinson et al., 2012; Bandau et al., 2015; Decker et al., 2016), a feature that was also observed in our transformed genotypes. CTs are considered to be anti-oxidant phenolic polymers (Gourlay and Constable, 2019) that are expected to influence the presence and impact of plant-consuming microorganisms and herbivores (Mutikainen et al., 2000; Bailey et al., 2005; Barbehenn and Constabel, 2011), although they also express a high degree of plasticity in response to environmental factors such as nitrogen addition (Bandau et al., 2015) and they may be equally important and indicative of the extent of internal recovery and the mode of growth (Harding et al., 2013; Lindroth and Madritch, 2015; Decker et al., 2016). However, the two lines in this experiment that suffered from elevated chewing symptoms varied in tannin content, with *35S:HjAXE* giving lower and *35SAnAXE1* higher foliar CT concentrations when compared to WT (Figures 5B,C). No consistent relationship between CTs and chewing damage caused could therefore be deduced from this study, supporting the hypothesis that the potential defensive role of CTs in plants is indeed complex.

Salicinoid phenolic glucosides have often been investigated as suggested markers of innate resistance to herbivore damage in woody species (Mutikainen et al., 2000; Albrectsen et al., 2004; Philippe and Bohlmann, 2007; Witzell and Martin, 2008; Fabisch et al., 2019) and in particular as constitutive markers in aspen (Albrectsen et al., 2010; Robinson et al., 2012; Bernhardsson et al., 2013; Lindroth and St. Clair, 2013; Lindroth et al., 2015). The *35S*-driven transgenes appeared to be more affected with respect to their SPG profiles compared to the *WP*-driven transgenes, although the low number of lines per construct tested in the present experiment (between two and four) resulted in few significant changes in SPG contents among the constructs (Table 2 and Supplementary Tables S2, S4). Despite this deficiency, the greater impact of the *35S* promoter compared to the *WP* promoter on SPG profiles was obvious. The synthesis of phenolic compounds belonging to the SPG group is still unresolved due to reticulate pathways with no apparent direct connection to the most simple salicinoid, salicin (Babst et al., 2010; Fellenberg et al., 2020), although it is increasingly accepted that the specialized metabolism of phenolic compounds is tightly linked to primary metabolism (Harding et al., 2013), and our study further suggests that cell wall acetylation may indeed alter, and determine levels of, phenolic compounds in aspen.

### CE1 and CE5 AXEs Induce Distinct Foliar Phenotypes

The selection of enzymes appropriate for transgenic modification was addressed in this study by comparing two fungal enzymes, a CE1 representative, *An*AXE1 and a CE5 representative, *Hj*AXE, expressed from either *35S* or *WP* promoters, for their effects on several traits related to growth, biotic stress resistance and foliage characteristics. Stem growth and leaf weight were not affected by the enzyme used. In contrast, the occurrence of necrosis was associated with *HjAXE*, and there was a higher incidence of chewing with *AnAXE1*, although both transgenes induced more chewing than was seen in WT (Figure 5 and Supplementary Table S6). CTs were more characteristic of *HjAXE* than *AnAXE1* expressing plants, and several SPGs accumulated differentially in the leaves of transgenic plants with the two transgenes (Figure 5 and Table 2). These differences were primarily seen in the lines with *35S*-driven transgenes; effects were negligible in lines with the WP promoter. These data indicate that each transgene induced different susceptibilities to specific biotic stresses, associated with different patterns of accumulation of some stress-related SPGs and CTs. Previous greenhouse studies with *35S:AnAXE1* and *35S:HjAXE* expressing plants did not reveal any major morphological differences between plants with the two transgenes (Ratke et al., 2015; Pawar et al., 2017a), highlighting the importance of field testing.

The physiological background behind the contrasting phenotypes observed in *35SAnAXE1* and *35SHjAXE* expressing plants is not known, and it could encompass many factors. Beside the difference in enzymatic specificities and mode of action in the cell wall (discussed by Wang et al., 2020), the two proteins could be differentially perceived by the pathogen-associated molecular patterns (PAMP) recognition system (Bellincampi et al., 2014). The induction of necrosis by ectopically expressed *HjAXE* seen in our trial (Figure 5) is reminiscent of the effects of several fungal xylanases from family 11, including *Hypocrea jecorina* xylanase II, which induce ethylene and hypersensitive responses in plants, leading to necrosis (Noda et al., 2010). A conserved amino acid motif, TEIGSVTSDGS, has been identified as being involved in induction of necrosis. Amino acid alignments of sequences used in the two constructs reveal that *Hj*AXE, but not *An*AXE1, includes a similar motif, 183-VGTCTTQG-190, and it would be interesting to test this for necrosis-inducing activity.

### Imbalance in Cellular Acetyl Levels Could Lead to Genomic Instability

In this trial, three out of 18 transgenic lines with reduced acetylation exhibited dwarfism (Figure 1B) and growth abnormalities (Figure 1C), and one of them showed increased mortality (Figure 1D). These detrimental effects could not be associated with the transgenes introduced, and they are likely to have been caused by somaclonal variation or positional effects. Such a high (17%) incidence of dwarfism in acetylation-modified lines is, however, remarkable, and it was not predicted on the basis of the growth observed during greenhouse trials with the same transgenic lines. We also observed no dwarfism other than dwarfism related to transgenes among another set of 48 transgenic lines modified for other qualities that were grown in nearby transgenic fields. Reports from previous American field trials support the conclusion that somaclonal variations or detrimental positional effects are rare in transgenic poplars. For example, in a long-term trial with 948 lines engineered for sterility, not a single incident of detectable somaclonal variation was reported (Klocko et al., 2018); similarly, in a survey of field trial studies in United States covering a period of over 20 years and more than 100 transgenic poplar lines, only 0.1–1% dwarfism that could putatively be linked to positional effects or somaclonal variation was detected (Strauss et al., 2016).

The exceptionally high occurrence of random dwarfism and abnormalities among our transgenic lines with reduced acetyl content suggests a potential link between the acetylation status and genomic stability. Indeed, studies in other eucaryotes including mammals and yeasts showed that cellular levels of acetyl-CoA are directly associated with histone acetylation, which in turn regulates chromatin epigenetic state (Cai and Tu, 2011; Etchegaray and Mostoslavsky, 2016). In plants, epigenetic changes in chromatin state have been linked to the activation of transposable elements under stress conditions, thus contributing to somaclonal variation (Kaeppler et al., 2000). Moreover, in mammalian cells, the cellular ability to repair double strand breaks in DNA requires histone acetylation (Sivanand et al., 2017). These data support the hypothesis that the higher levels of acetyl-CoA expected to be induced by our engineering strategies could indeed lead to increased rates of mutation, especially when combined with stress. This hypothesis could be addressed by field testing and whole-genome sequencing of different acetyl-modified plants.

## Concluding Remarks

The field growth of genetically modified plants is highly controversial in large parts of the World, but it is also warranted so that their potential can be carefully validated (Strauss, 2003; Viswanath et al., 2012; Strauss et al., 2016). On the one hand, genetic modifications promise to make it possible to tailor plants to perform better and produce higher quality products. On the other hand, genetic modification is a contentious topic among the public. The only way to assess the benefits and drawbacks of applying genetic modification techniques to crops, including forest trees, is to perform thorough characterization of transgenic lines. Here we present the results of the first field test of transgenic plants that target xylan-acetylation in cell walls, carried out to assess the consequences for growth, environmental stress resistance and biotic stress resistance in conditions similar to those used in short-rotation plantation forestry. Our data revealed novel plant phenotypes, not seen in the previous greenhouse experiments, as well as novel traits concerning the interaction of the genetically modified trees with their environment. The results highlight the need for early field testing in order to evaluate transgenic strategy and to assess the potential benefits and drawbacks expected when transgenic crops are used compared to their non-transgenic commercial counterparts.

## Data Availability Statement

All datasets generated for this study are included in the article/Supplementary Material.

## Author Contributions

MD-M, BA, and EM designed the research. MD-M, FA, ED, LM, UJ, BA, and EM carried out field the work and sample preparation. PP produced the transgenic lines. FA conducted the tannin analyses and prepared the leaves for metabolomics analyses. MD-M, ED, BA, and EM analyzed the data. BA and EM wrote the manuscript with contributions from all authors.

## Conflict of Interest

The authors declare that the research was conducted in the absence of any commercial or financial relationships that could be construed as a potential conflict of interest.

## Abbreviations

*35S*: Cauliflower mosaic virus 3*5S* promoter
ABA: abscisic acid
*An*AXE1: *Aspergillus niger* ACETYL XYLAN ESTERASE 1
CT: Condensed Tannin
*Hj*AXE: *Hypocrea jecorina* ACETYL XYLAN ESTERASE
IAA: indole-3-acetic acid
JA: jasmonic acid
PAE9: PECTATE ACETYLESTERASE 9
RGI: rhamnogalacturonan I
RWA: REDUCED WALL ACETYLATION
SA: salicylic acid
SPGs: salicinoid phenolic glucosides
TBL: TRICHOME BIREFRINGENCY-LIKE
*WP*: Wood Promoter.
