# Supplementary material for: Cell Wall Acetylation in Hybrid Aspen Affects Field Performance, Foliar Phenolic Composition and Resistance to Biological Stress Factors in a Construct-Dependent Fashion
Source: Front Plant Sci. 2020 May 25;11:651. doi: 10.3389/fpls.2020.00651 (PMC7265884; doi:10.3389/fpls.2020.00651)
Supplement: Supplementary file 2 [file Presentation_1.pptx]

## Slide 1
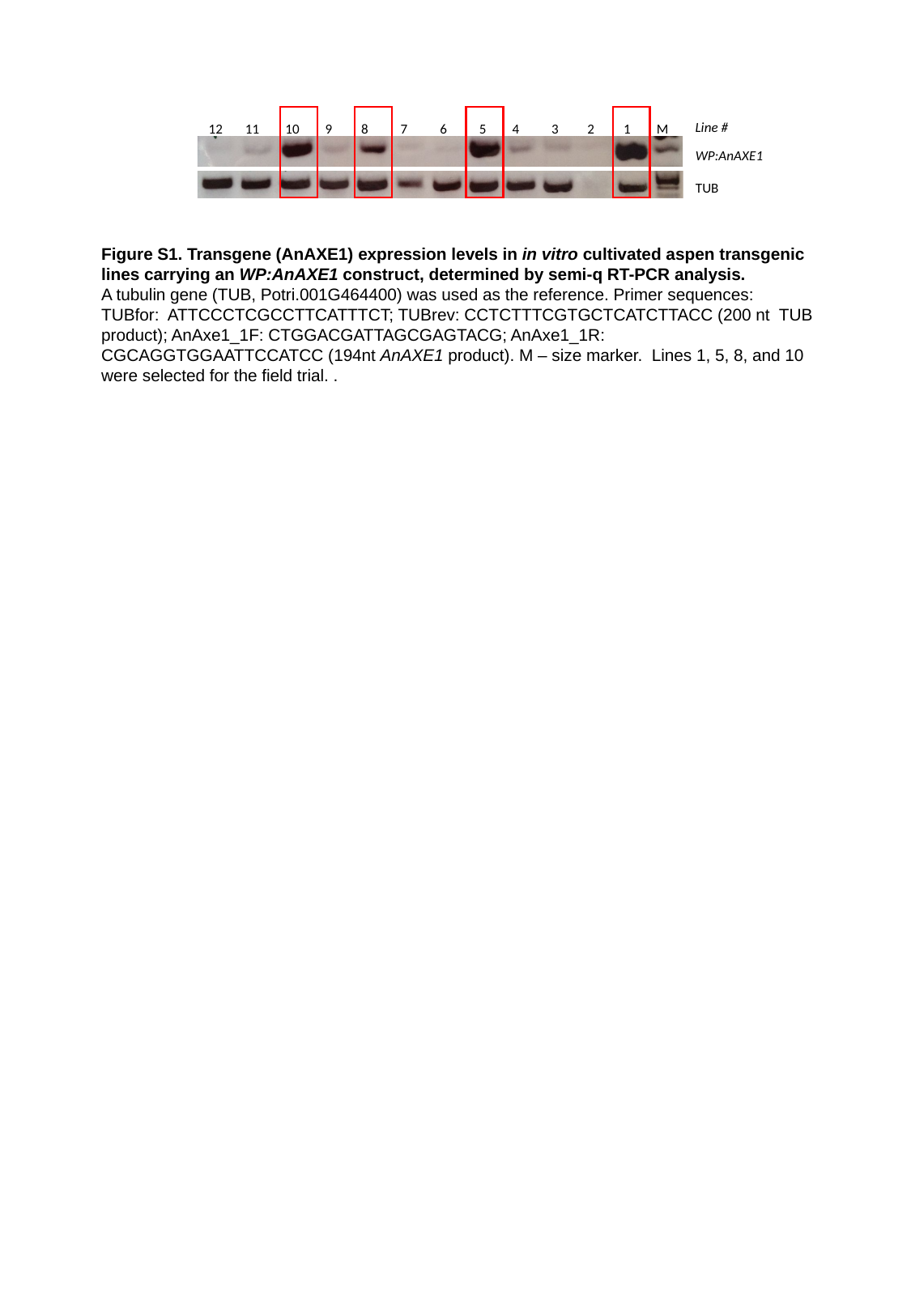

Line #
12 11 10 9 8 7 6 5 4 3 2 1 M
WP:AnAXE1
TUB
Figure S1. Transgene (AnAXE1) expression levels in in vitro cultivated aspen transgenic lines carrying an WP:AnAXE1 construct, determined by semi-q RT-PCR analysis.
A tubulin gene (TUB, Potri.001G464400) was used as the reference. Primer sequences: TUBfor: ATTCCCTCGCCTTCATTTCT; TUBrev: CCTCTTTCGTGCTCATCTTACC (200 nt TUB product); AnAxe1_1F: CTGGACGATTAGCGAGTACG; AnAxe1_1R: CGCAGGTGGAATTCCATCC (194nt AnAXE1 product). M – size marker. Lines 1, 5, 8, and 10 were selected for the field trial. .

## Slide 2
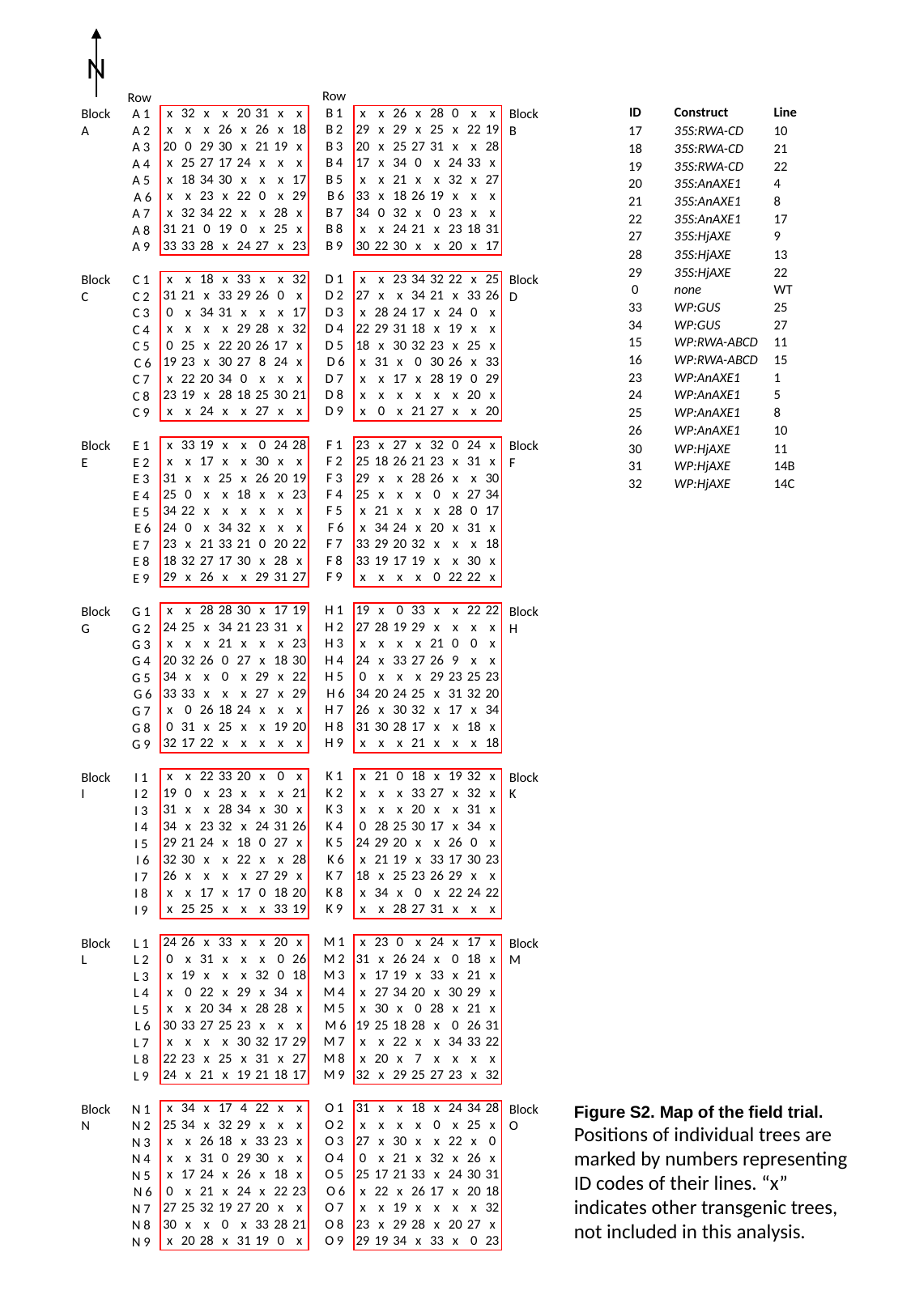

N
| | | | | | | | | | | | | | | | | | | | | | |
| --- | --- | --- | --- | --- | --- | --- | --- | --- | --- | --- | --- | --- | --- | --- | --- | --- | --- | --- | --- | --- | --- |
| | Row | | | | | | | | | | Row | | | | | | | | | | |
| Block | A 1 | x | 32 | x | x | 20 | 31 | x | x | | B 1 | x | x | 26 | x | 28 | 0 | x | x | | Block |
| A | A 2 | x | x | x | 26 | x | 26 | x | 18 | | B 2 | 29 | x | 29 | x | 25 | x | 22 | 19 | | B |
| | A 3 | 20 | 0 | 29 | 30 | x | 21 | 19 | x | | B 3 | 20 | x | 25 | 27 | 31 | x | x | 28 | | |
| | A 4 | x | 25 | 27 | 17 | 24 | x | x | x | | B 4 | 17 | x | 34 | 0 | x | 24 | 33 | x | | |
| | A 5 | x | 18 | 34 | 30 | x | x | x | 17 | | B 5 | x | x | 21 | x | x | 32 | x | 27 | | |
| | A 6 | x | x | 23 | x | 22 | 0 | x | 29 | | B 6 | 33 | x | 18 | 26 | 19 | x | x | x | | |
| | A 7 | x | 32 | 34 | 22 | x | x | 28 | x | | B 7 | 34 | 0 | 32 | x | 0 | 23 | x | x | | |
| | A 8 | 31 | 21 | 0 | 19 | 0 | x | 25 | x | | B 8 | x | x | 24 | 21 | x | 23 | 18 | 31 | | |
| | A 9 | 33 | 33 | 28 | x | 24 | 27 | x | 23 | | B 9 | 30 | 22 | 30 | x | x | 20 | x | 17 | | |
| | | | | | | | | | | | | | | | | | | | | | |
| Block | C 1 | x | x | 18 | x | 33 | x | x | 32 | | D 1 | x | x | 23 | 34 | 32 | 22 | x | 25 | | Block |
| C | C 2 | 31 | 21 | x | 33 | 29 | 26 | 0 | x | | D 2 | 27 | x | x | 34 | 21 | x | 33 | 26 | | D |
| | C 3 | 0 | x | 34 | 31 | x | x | x | 17 | | D 3 | x | 28 | 24 | 17 | x | 24 | 0 | x | | |
| | C 4 | x | x | x | x | 29 | 28 | x | 32 | | D 4 | 22 | 29 | 31 | 18 | x | 19 | x | x | | |
| | C 5 | 0 | 25 | x | 22 | 20 | 26 | 17 | x | | D 5 | 18 | x | 30 | 32 | 23 | x | 25 | x | | |
| | C 6 | 19 | 23 | x | 30 | 27 | 8 | 24 | x | | D 6 | x | 31 | x | 0 | 30 | 26 | x | 33 | | |
| | C 7 | x | 22 | 20 | 34 | 0 | x | x | x | | D 7 | x | x | 17 | x | 28 | 19 | 0 | 29 | | |
| | C 8 | 23 | 19 | x | 28 | 18 | 25 | 30 | 21 | | D 8 | x | x | x | x | x | x | 20 | x | | |
| | C 9 | x | x | 24 | x | x | 27 | x | x | | D 9 | x | 0 | x | 21 | 27 | x | x | 20 | | |
| | | | | | | | | | | | | | | | | | | | | | |
| Block | E 1 | x | 33 | 19 | x | x | 0 | 24 | 28 | | F 1 | 23 | x | 27 | x | 32 | 0 | 24 | x | | Block |
| E | E 2 | x | x | 17 | x | x | 30 | x | x | | F 2 | 25 | 18 | 26 | 21 | 23 | x | 31 | x | | F |
| | E 3 | 31 | x | x | 25 | x | 26 | 20 | 19 | | F 3 | 29 | x | x | 28 | 26 | x | x | 30 | | |
| | E 4 | 25 | 0 | x | x | 18 | x | x | 23 | | F 4 | 25 | x | x | x | 0 | x | 27 | 34 | | |
| | E 5 | 34 | 22 | x | x | x | x | x | x | | F 5 | x | 21 | x | x | x | 28 | 0 | 17 | | |
| | E 6 | 24 | 0 | x | 34 | 32 | x | x | x | | F 6 | x | 34 | 24 | x | 20 | x | 31 | x | | |
| | E 7 | 23 | x | 21 | 33 | 21 | 0 | 20 | 22 | | F 7 | 33 | 29 | 20 | 32 | x | x | x | 18 | | |
| | E 8 | 18 | 32 | 27 | 17 | 30 | x | 28 | x | | F 8 | 33 | 19 | 17 | 19 | x | x | 30 | x | | |
| | E 9 | 29 | x | 26 | x | x | 29 | 31 | 27 | | F 9 | x | x | x | x | 0 | 22 | 22 | x | | |
| | | | | | | | | | | | | | | | | | | | | | |
| Block | G 1 | x | x | 28 | 28 | 30 | x | 17 | 19 | | H 1 | 19 | x | 0 | 33 | x | x | 22 | 22 | | Block |
| G | G 2 | 24 | 25 | x | 34 | 21 | 23 | 31 | x | | H 2 | 27 | 28 | 19 | 29 | x | x | x | x | | H |
| | G 3 | x | x | x | 21 | x | x | x | 23 | | H 3 | x | x | x | x | 21 | 0 | 0 | x | | |
| | G 4 | 20 | 32 | 26 | 0 | 27 | x | 18 | 30 | | H 4 | 24 | x | 33 | 27 | 26 | 9 | x | x | | |
| | G 5 | 34 | x | x | 0 | x | 29 | x | 22 | | H 5 | 0 | x | x | x | 29 | 23 | 25 | 23 | | |
| | G 6 | 33 | 33 | x | x | x | 27 | x | 29 | | H 6 | 34 | 20 | 24 | 25 | x | 31 | 32 | 20 | | |
| | G 7 | x | 0 | 26 | 18 | 24 | x | x | x | | H 7 | 26 | x | 30 | 32 | x | 17 | x | 34 | | |
| | G 8 | 0 | 31 | x | 25 | x | x | 19 | 20 | | H 8 | 31 | 30 | 28 | 17 | x | x | 18 | x | | |
| | G 9 | 32 | 17 | 22 | x | x | x | x | x | | H 9 | x | x | x | 21 | x | x | x | 18 | | |
| | | | | | | | | | | | | | | | | | | | | | |
| Block | I 1 | x | x | 22 | 33 | 20 | x | 0 | x | | K 1 | x | 21 | 0 | 18 | x | 19 | 32 | x | | Block |
| I | I 2 | 19 | 0 | x | 23 | x | x | x | 21 | | K 2 | x | x | x | 33 | 27 | x | 32 | x | | K |
| | I 3 | 31 | x | x | 28 | 34 | x | 30 | x | | K 3 | x | x | x | 20 | x | x | 31 | x | | |
| | I 4 | 34 | x | 23 | 32 | x | 24 | 31 | 26 | | K 4 | 0 | 28 | 25 | 30 | 17 | x | 34 | x | | |
| | I 5 | 29 | 21 | 24 | x | 18 | 0 | 27 | x | | K 5 | 24 | 29 | 20 | x | x | 26 | 0 | x | | |
| | I 6 | 32 | 30 | x | x | 22 | x | x | 28 | | K 6 | x | 21 | 19 | x | 33 | 17 | 30 | 23 | | |
| | I 7 | 26 | x | x | x | x | 27 | 29 | x | | K 7 | 18 | x | 25 | 23 | 26 | 29 | x | x | | |
| | I 8 | x | x | 17 | x | 17 | 0 | 18 | 20 | | K 8 | x | 34 | x | 0 | x | 22 | 24 | 22 | | |
| | I 9 | x | 25 | 25 | x | x | x | 33 | 19 | | K 9 | x | x | 28 | 27 | 31 | x | x | x | | |
| | | | | | | | | | | | | | | | | | | | | | |
| Block | L 1 | 24 | 26 | x | 33 | x | x | 20 | x | | M 1 | x | 23 | 0 | x | 24 | x | 17 | x | | Block |
| L | L 2 | 0 | x | 31 | x | x | x | 0 | 26 | | M 2 | 31 | x | 26 | 24 | x | 0 | 18 | x | | M |
| | L 3 | x | 19 | x | x | x | 32 | 0 | 18 | | M 3 | x | 17 | 19 | x | 33 | x | 21 | x | | |
| | L 4 | x | 0 | 22 | x | 29 | x | 34 | x | | M 4 | x | 27 | 34 | 20 | x | 30 | 29 | x | | |
| | L 5 | x | x | 20 | 34 | x | 28 | 28 | x | | M 5 | x | 30 | x | 0 | 28 | x | 21 | x | | |
| | L 6 | 30 | 33 | 27 | 25 | 23 | x | x | x | | M 6 | 19 | 25 | 18 | 28 | x | 0 | 26 | 31 | | |
| | L 7 | x | x | x | x | 30 | 32 | 17 | 29 | | M 7 | x | x | 22 | x | x | 34 | 33 | 22 | | |
| | L 8 | 22 | 23 | x | 25 | x | 31 | x | 27 | | M 8 | x | 20 | x | 7 | x | x | x | x | | |
| | L 9 | 24 | x | 21 | x | 19 | 21 | 18 | 17 | | M 9 | 32 | x | 29 | 25 | 27 | 23 | x | 32 | | |
| | | | | | | | | | | | | | | | | | | | | | |
| Block | N 1 | x | 34 | x | 17 | 4 | 22 | x | x | | O 1 | 31 | x | x | 18 | x | 24 | 34 | 28 | | Block |
| N | N 2 | 25 | 34 | x | 32 | 29 | x | x | x | | O 2 | x | x | x | x | 0 | x | 25 | x | | O |
| | N 3 | x | x | 26 | 18 | x | 33 | 23 | x | | O 3 | 27 | x | 30 | x | x | 22 | x | 0 | | |
| | N 4 | x | x | 31 | 0 | 29 | 30 | x | x | | O 4 | 0 | x | 21 | x | 32 | x | 26 | x | | |
| | N 5 | x | 17 | 24 | x | 26 | x | 18 | x | | O 5 | 25 | 17 | 21 | 33 | x | 24 | 30 | 31 | | |
| | N 6 | 0 | x | 21 | x | 24 | x | 22 | 23 | | O 6 | x | 22 | x | 26 | 17 | x | 20 | 18 | | |
| | N 7 | 27 | 25 | 32 | 19 | 27 | 20 | x | x | | O 7 | x | x | 19 | x | x | x | x | 32 | | |
| | N 8 | 30 | x | x | 0 | x | 33 | 28 | 21 | | O 8 | 23 | x | 29 | 28 | x | 20 | 27 | x | | |
| | N 9 | x | 20 | 28 | x | 31 | 19 | 0 | x | | O 9 | 29 | 19 | 34 | x | 33 | x | 0 | 23 | | |
| ID | Construct | Line |
| --- | --- | --- |
| 17 | 35S:RWA-CD | 10 |
| 18 | 35S:RWA-CD | 21 |
| 19 | 35S:RWA-CD | 22 |
| 20 | 35S:AnAXE1 | 4 |
| 21 | 35S:AnAXE1 | 8 |
| 22 | 35S:AnAXE1 | 17 |
| 27 | 35S:HjAXE | 9 |
| 28 | 35S:HjAXE | 13 |
| 29 | 35S:HjAXE | 22 |
| 0 | none | WT |
| 33 | WP:GUS | 25 |
| 34 | WP:GUS | 27 |
| 15 | WP:RWA-ABCD | 11 |
| 16 | WP:RWA-ABCD | 15 |
| 23 | WP:AnAXE1 | 1 |
| 24 | WP:AnAXE1 | 5 |
| 25 | WP:AnAXE1 | 8 |
| 26 | WP:AnAXE1 | 10 |
| 30 | WP:HjAXE | 11 |
| 31 | WP:HjAXE | 14B |
| 32 | WP:HjAXE | 14C |
Figure S2. Map of the field trial.
Positions of individual trees are marked by numbers representing ID codes of their lines. “x” indicates other transgenic trees, not included in this analysis.

## Slide 3
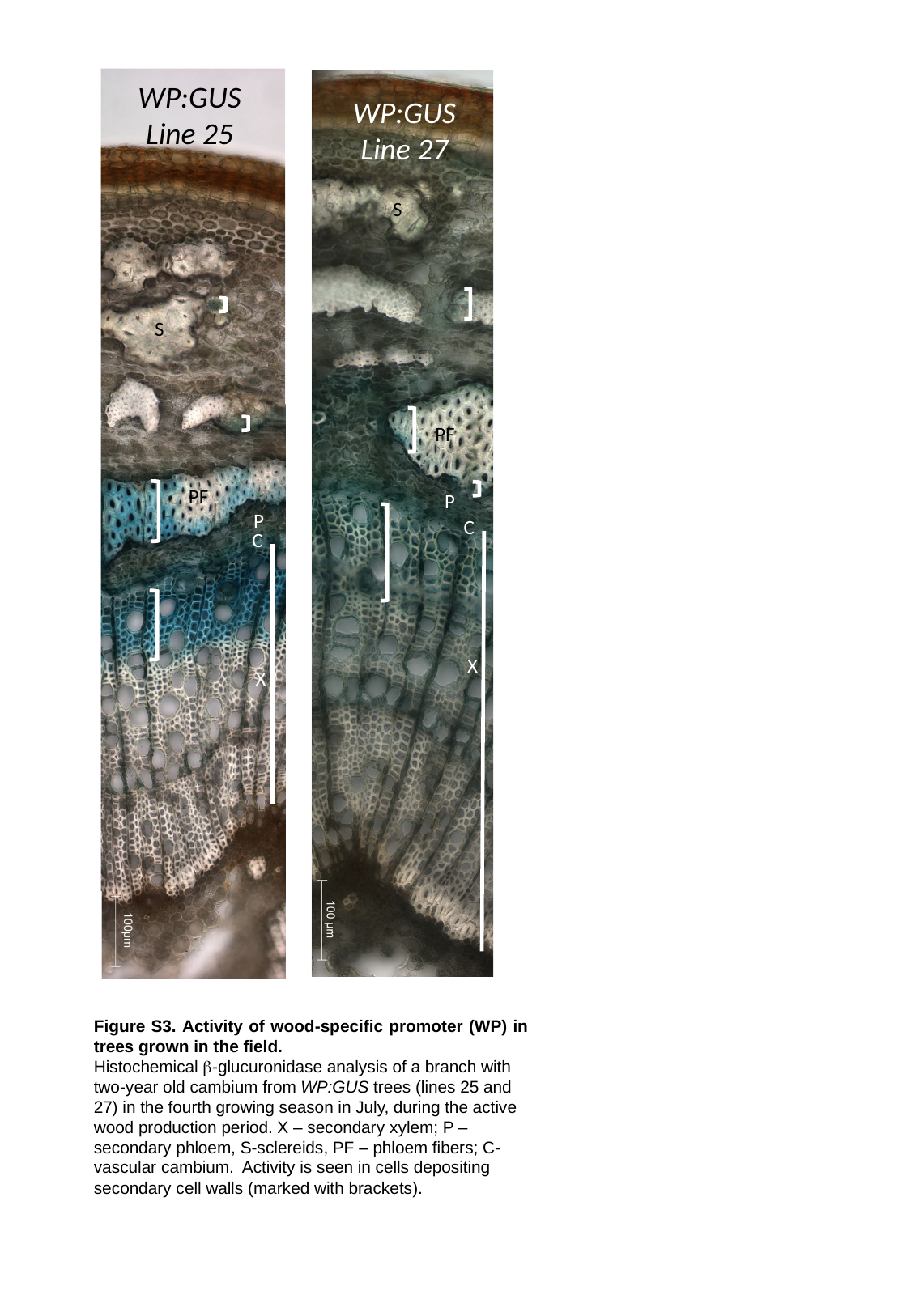

WP:GUS
Line 25
S
PF
P
C
X
WP:GUS
Line 27
S
PF
P
C
X
Figure S3. Activity of wood-specific promoter (WP) in trees grown in the field.
Histochemical b-glucuronidase analysis of a branch with two-year old cambium from WP:GUS trees (lines 25 and 27) in the fourth growing season in July, during the active wood production period. X – secondary xylem; P – secondary phloem, S-sclereids, PF – phloem fibers; C-vascular cambium. Activity is seen in cells depositing secondary cell walls (marked with brackets).
